# Theoretical and Experimental Studies on MEMS Variable Cross-Section Cantilever Beam Based Piezoelectric Vibration Energy Harvester

**DOI:** 10.3390/mi12070772

**Published:** 2021-06-30

**Authors:** Xianming He, Dongxiao Li, Hong Zhou, Xindan Hui, Xiaojing Mu

**Affiliations:** 1 Key Laboratory of Optoelectronic Technology & Systems, Ministry of Education, Chongqing University, Chongqing 400044, China; lidongxiao@cqu.edu.cn (D.L.); zhlf@cqu.edu.cn (H.Z.); huixindan@cqu.edu.cn (X.H.); 2 International R & D Center of Micro-Nano Systems and New Materials Technology, Chongqing University, Chongqing 400044, China

**Keywords:** piezoelectric vibration energy harvester, variable cross-section cantilever beam, MEMS, trapezoidal cantilever beam, coupled distributed parameter dynamics model, AlN

## Abstract

The piezoelectric vibration energy harvester (PVEH) based on the variable cross-section cantilever beam (VCSCB) structure has the advantages of uniform axial strain distribution and high output power density, so it has become a research hotspot of the PVEH. However, its electromechanical model needs to be further studied. In this paper, the bidirectional coupled distributed parameter electromechanical model of the MEMS VCSCB based PVEH is constructed, analytically solved, and verified, which laid an important theoretical foundation for structural design and optimization, performance improvement, and output prediction of the PVEH. Based on the constructed model, the output performances of five kinds of VCSCB based PVEHs with different cross-sectional shapes were compared and analyzed. The results show that the PVEH with the concave quadratic beam shape has the best output due to the uniform surface stress distribution. Additionally, the influence of the main structural parameters of the MEMS trapezoidal cantilever beam (TCB) based PVEH on the output performance of the device is theoretically analyzed. Finally, a prototype of the Aluminum Nitride (AlN) TCB based PVEH is designed and developed. The peak open-circuit voltage and normalized power density of the device can reach 5.64 V and 742 μW/cm^3^/g^2^, which is in good agreement with the theoretical model value. The prototype has wide application prospects in the power supply of the wireless sensor network node such as the structural health monitoring system and the Internet of Things.

## 1. Introduction

In recent years, the rapid growth of smart cities, automotive electronics, and mobile communications has promoted the rapid development of wireless sensor networks and portable electronic devices. However, the long-lived, integrated, and miniaturized development of wireless sensor network nodes and portable electronic devices pose new challenges to their energy supply units. The new challenges place higher demands on energy supply units, such as small size, long life, high output power density, easy monolithic integration, and resistance to fail in harsh environments, etc. Micro-environment energy harvesting technology is a forward-looking enabling technology for low-power wireless sensor network nodes and portable electronic devices to provide durable and reliable power. The MEMS piezoelectric vibration energy harvester (PVEH) can use piezoelectric materials to continuously convert the almost everywhere vibration energy in the environment into electrical energy. Due to its obvious advantages such as no electromagnetic interference, good MEMS process compatibility, high output power density, high reliability, and mass production, it has become the research focus of energy harvesting technology.

Currently, researchers have carried out extensive research on the theoretical models, materials, and structures of MEMS PVEHs. The theoretical models are mainly divided into two categories: lumped parameter (LP) models [1,2,3] and distributed parameter (DP) models [4,5,6]. The research of materials mainly focuses on substrate materials [7,8], doping optimization [9,10] modification, and new piezoelectric materials [11,12]. Aluminum Nitride (AlN) piezoelectric film is an important energy-trapping film for the MEMS PVEH, and its doping modification has become a research hotspot. The structural design mainly focuses on optimizing traditional structures and proposing new structures. The cantilever structure is the most traditional and widely studied one. The research on cantilever beam-based PVEHs mainly focuses on stress optimization [13], geometric shape optimization [14], electrode optimization [15], topology optimization [16], and frequency band expansion [17,18], etc.

Although MEMS PVEHs have been rapidly developed in terms of theoretical models, materials, and structures, the research mainly focuses on the rectangular cantilever beam. Since the bending stress borne by the rectangular cantilever beam varies linearly along the length, the bending stress is maximized at the fixed end and tends to be zero at the free tip. Therefore, the rectangular cantilever beam PVEH may have problems; excessive strain and fatigue may occur around the fixed end, and the piezoelectric material around the free tip does not play its full role. In recent years, researchers began to study variable cross-section cantilever beam (VCSCB) based piezoelectric energy harvesters. Baker et al. [19] pointed out that changing the beam’s cross-section linearly along its length increases the energy harvesters’ output power density up to 30%. Salmani and Rahimi [20] proposed an exact analytical solution to simulate an exponentially tapered energy harvester’s dynamical and electrical behavior. The results show that exponential tapering may lead to extract more voltage and power per mass of the piezoelectric energy harvester. To minimize the stress non-uniformity in conventional cantilever-based PVEH, Kundu et al. [21] proposed a bimorph cantilever PVEH with a thickness tapered geometry, and the analytical expressions for the displacement, stress, and generated voltage are derived for sinusoidal input excitations. The results show that the proposed thickness-tapered PVEH generates 20% more power than the equivalent conventional PVEH with uniform thickness. Umegaki et al. [22] constructed an equivalent circuit model of the PVEH which is suitable for the trapezoidal piezoelectric unimorph or bimorph cantilevers. The theoretical formula of output power can not only optimize the geometry of the trapezoidal cantilever, but also optimize the electrode length and substrate thickness. Zhang et al. [23] established a theoretical model of a trapezoidal beam PVEH, and derived the corresponding calculation formulas of the resonant frequency, the voltage, and the power. Salmani et al. [24] proposed an accurate analytical solution to calculate the power generated by the vibration of exponentially tapered unimorph and bimorph with series and parallel connections. Khazaee et al. [25] analyzed the influence of the taper angle of the trapezoidal cantilever beam on the output power and natural frequency. The results show that the taper angle, as a key parameter to change the natural frequency of the PVEH, can be used for broadband energy harvesting by the PVEH. At the same time, the taper angle causes a significant increase in power density. In addition to the tapered width, varying thickness is another practical method to improve the output power of the PVEHs. Khazaee et al. [26] proposed a comprehensive finite element formulation for accurate structure and energy harvesting modeling of piezoelectric beams. The proposed high-order shear FEM is suitable for thick composite-based non-uniform PVEHs. The results show that increasing the piezoelectric thickness along the length of the beam can increase the piezoelectric output power. Xie et al. [27] proposed a cantilever PVEH that is tapered in width and thickness, and developed a corresponding finite differential model to simulate the tapered energy harvester for estimating its efficiency by examining a governing differential equation with variable coefficients. Hajheidari et al. [28] proposed a numerical solution for the tapered energy harvesting beam with different degrees of nonuniformity and tapering parameter. The width and height of the cantilever beam of the device vary according to the degree of the polynomial function. The results show that it is feasible to increase the power output of the PVEH by increasing the degree of non-uniformity and slenderness ratio. In short, researchers have conducted much research on the electromechanical model construction and performance analysis of the tapered piezoelectric vibration energy harvester, especially the trapezoidal beam PVEH. The tapered beam PVEH has advantages in stress optimization, frequency adjustment, and performance improvement. However, the analytical solution and approximate solution method of the electromechanical model of the tapered beam PVEH in the existing literature is more complicated, and the solution is not easy to numerically analyze. At the same time, it lacks a bidirectional coupled distributed parameter electromechanical model suitable for a MEMS variable cross-section beam PVEH of arbitrary shape.

Herein, in this paper, we construct, analytically solve and verify the bidirectional coupled distributed parameter dynamics model of the MEMS VCSCB based PVEH. Additionally, the mapping relationship and influence law of the structural and material parameters on its normalized output power density are obtained, which laid an important theoretical foundation for structural design and optimization, performance improvement, and output prediction of the MEMS PVEH. The proposed analytical model is suitable for the cantilever beam based PVEH with arbitrary cross-sectional shapes in d31 mode. It has the advantages of high prediction accuracy, fast calculation speed, availability of an analytical solution, and it can effectively solve the problem that the existing model can only be applied to the regular-shaped cantilever beam based PVEH. Based on the constructed model, the output performances of five kinds of VCSCB based PVEHs with different cross-sectional shapes were compared and analyzed, and the influence of the main structural parameters on the output performance of the device were theoretically analyzed. Finally, a prototype of the AlN trapezoidal cantilever beam (TCB) based PVEH was developed and was used to experimentally verify the proposed theoretical model.

## 2. Theoretical Model

**Figure 1:**
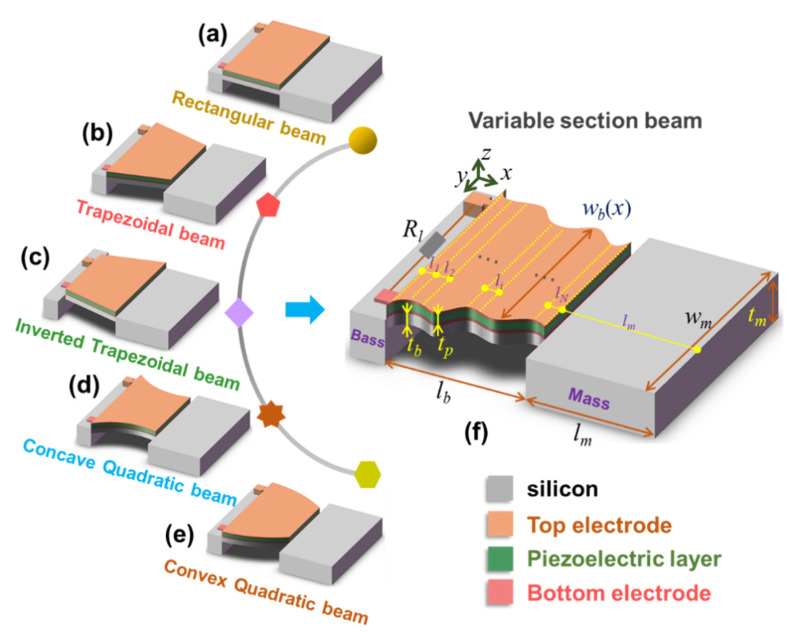
Structure diagrams of several common MEMS variable cross-section cantilever beam based piezoelectric vibration energy harvesters, (**a**) rectangular beam type, (**b**) trapezoidal beam type, (**c**) inverted trapezoidal beam type, (**d**) concave quadratic beam type, (**e**) convex quadratic beam type, and (**f**) variable section beam type.

The structure diagrams of several common MEMS VCSCB based PVEHs are shown in Figure 1; they are mainly composed of the base, the Si beam, the Si mass, the piezoelectric layer, and the top and bottom electrodes. To improve the normalized output power density of the MEMS PVEH, it is often necessary to optimize the key structural parameters of the device according to the characteristics of a given environmental vibration source. However, the optimal design needs to be based on the dynamic electromechanical model. Next, we will construct and solve the dynamic electromechanical coupling model of the MEMS PVEH with any cross-sectional shape (Figure 1f), and the derived model is also suitable for common MEMS VCSCB based PVEHs such as the rectangular beam type (Figure 1a), trapezoidal beam types (Figure 1b,c), and quadratic beam types (Figure 1d,e).

The main structural parameters of the device are shown in Figure 1f. Assume that the axial direction is the *x*-axis, and the thickness direction is the *z*-axis. $l_{b}$, $t_{b}$, $l_{m}$, $t_{m}$ and $t_{p}$ respectively represent the beam length, the beam thickness, the mass length, the mass thickness, and the piezoelectric layer thickness of the VCSCB, respectively. The widths of the VCSCB and the tip mass are $w_{b}(x)$ and $w_{m}$, respectively. Let $\rho_{b}$, $\rho_{p}$, and $\rho_{m}$ denote the density of the beam substrate, the piezoelectric layer, and the tip mass, respectively. Let $Y_{b}$, $Y_{p}$, and $Y_{m}$ denote Young’s elastic modulus of the beam substrate, the piezoelectric layer, and the tip mass, respectively. m(x) and $M_{m}$ are the mass line density of the VCSCB and the tip mass, respectively. $c_{a}(x)$ and $c_{s}(x)$ are the air viscous damping coefficient (external damping coefficient) and the material strain rate damping coefficient (internal damping coefficient) of the VCSCB, respectively, and ${c^{\prime }}_{a}(x)$ ${c^{\prime }}_{s}(x)$ are those of the tip mass. $e_{31}$, $\varepsilon_{{}_{33}}^{s}$, and $R_{l}$ are the effective piezoelectric stress constant, the dielectric constant of the piezoelectric material under constant strain, and the external load resistance, respectively.

Because the MEMS VCSCB based PVEH can ignore the influence of the shear deformation and the moment of inertia of the beam, the VCSCB can be treated as a Euler-Bernoulli beam. The thickness of the electrode is much smaller than the thickness of the piezoelectric layer and the substrate. The material is all isotropic, and the nonlinear behavior of the material is ignored. Therefore, the bending moments of the VCSCB and the tip mass bending at ***x*** are:

(1) $$\left\{ \begin{array}{l} M_{b}(x,t)=w_{b}(x)\left[Y_{b}\int_{-t_{b}+z_{N}}^{z_{N}}zT_{1}^{b}(x,z,t)dz+Y_{p}\int_{z_{N}}^{t_{p}+z_{N}}zT_{1}^{p}(x,z,t)dz\right]\\ M_{m}(x,t)=w_{m}\left[Y_{m}\int_{-t_{m}/2}^{t_{m}/2}zT_{1}^{m}(x,z,t)dz\right] \end{array}\right.$$

where $z_{N}$ is the coordinates of the neutral plane, and $T_{1}^{b}(x,z,t)$, $T_{1}^{p}(x,z,t)$, and $T_{1}^{m}(x,z,t)$ respectively represent the axial stress component of any point on the substrate layer, the piezoelectric layer, and the tip mass. Assuming that the corresponding axial strain components are $S_{1}^{b}(x,z,t)$, $S_{1}^{p}(x,z,t)$, and $S_{1}^{m}(x,z,t)$, and considering the internal damping of the material, the stress–strain relationship of each layer is as follows.

(2) $$\left\{ \begin{array}{l} T_{1}^{b}(x,z,t)=Y_{b}S_{1}^{b}(x,z,t)+c_{s}(x)\frac{\partial }{\partial t}S_{1}^{b}(x,z,t)\\ T_{1}^{p}(x,z,t)=Y_{p}S_{1}^{p}(x,z,t)-e_{31}E_{3}(t)+c_{s}(x)\frac{\partial }{\partial t}S_{1}^{p}(x,z,t)\\ T_{1}^{m}(x,z,t)=Y_{m}S_{1}^{m}(x,z,t)+{c^{\prime }}_{s}(x)\frac{\partial }{\partial t}S_{1}^{m}(x,z,t) \end{array}\right.$$

If the basic excitation of the device is $B(t)=B_{0}e^{i\Omega t}$, use z(x,t) to represent the lateral displacement of any point on the neutral axis of the beam relative to the clamping end. Combining the beam disturbance equation, the piezoelectric constitutive equation, the equivalent bending moment of the piezoelectric layer produced by the inverse piezoelectric effect, and the relationship between the axial strain and the curvature of the beam at this point, the Formula (1) can be rewritten as follows.

(3) $$\left\{ \begin{array}{l} M_{b}(x,t)=YI(x)\frac{\partial^{2}z(x,t)}{\partial x^{2}}+c_{s}(x)I_{b}(x)\frac{\partial^{3}z(x,t)}{\partial x^{3}\partial t}+\chi V(t)\left[H(x)-H(x-l_{b})\right]\\ M_{m}(x,t)=Y_{m}I_{m}\frac{\partial^{2}z(x,t)}{\partial x^{2}}+{c^{\prime }}_{s}(x)I_{m}\frac{\partial^{3}z(x,t)}{\partial x^{3}\partial t} \end{array}\right.$$

where $I_{b}(x)$ and $I_{m}$ are the moments of inertia of the VCSCB and the tip mass, respectively. YI(x) and $Y_{m}I_{m}$ represent the bending stiffness of the VCSCB and the tip mass, respectively. χ is the piezoelectric coupling term, *H(x)* is the Heaviside function, and *V(t)* is the output voltage of the device. Considering damping and combining the Euler-Bernoulli beam theory, the vibration differential equation of the device can be obtained. Combining the equivalent circuit of the device, the Gaussian formula, and piezoelectric constitutive equation, the coupling circuit equation of the device can be obtained [6]. Therefore, the bidirectional coupled distributed parameter dynamic model of the device based on the Euler-Bernoulli beam theory is as follows.

(4) $$\left\{ \begin{array}{l} \frac{\partial^{2}M_{b}(x,t)}{\partial x^{2}}+c_{a}(x)\frac{\partial z(x,t)}{\partial t}+m(x)\frac{\partial^{2}z(x,t)}{\partial t^{2}}=-m(x)\frac{\partial^{2}B(x,t)}{\partial t^{2}}-c_{a}(x)\frac{\partial B(x,t)}{\partial t}\begin{array}{c} \begin{array}{c} \begin{array}{cc} , & 0\leq x\leq l_{b} \end{array} \end{array} \end{array}\\ \frac{\partial^{2}M_{m}(x,t)}{\partial x^{2}}+{c^{\prime }}_{a}(x)\frac{\partial z(x,t)}{\partial t}+M_{m}\frac{\partial^{2}z(x,t)}{\partial t^{2}}=-M_{m}\frac{\partial^{2}B(x,t)}{\partial t^{2}}-{c^{\prime }}_{a}(x)\frac{\partial B(x,t)}{\partial t}\begin{array}{cc} , & l_{b}<x\leq l_{b}+l_{m} \end{array}\\ \frac{\varepsilon_{33}^{S}\int_{x=0}^{l_{b}}w_{b}(x)dx}{t_{p}}\frac{dV(t)}{dt}+\frac{V(t)}{R_{l}}+e_{31}\left(\frac{t_{p}}{2}+z_{N}\right)\int_{x=0}^{l_{b}}w_{b}(x)\frac{\partial z^{3}(x,t)}{\partial x^{2}\partial t}dx=0 \end{array}\right.$$

Next, the constructed DP model is solved by modal analysis and harmonic response analysis. Since the bending stiffness and the mass line density of the VCSCB are no longer constants, the analytical solution of the vibration equation cannot be solved directly by the method of separating variables. This paper adopts the infinitesimal method to divide the VCSCB into *N* section microbeams. If *N* is large enough, each microbeam can be regarded as a micro rectangular beam. At this time, the device can be divided into *N* section micro rectangular beams and a tip mass beam, giving a total of *N+1* section beams. The length, the bending stiffness, and the mass linear density of the *i*th microbeam are denoted by $l_{i}$, ${\left(YI\right)}_{i}$, and $m_{i}$, respectively. Thus, $l_{i}=l_{0}=l_{b}/N{,}_{}(i=1,2,3,\cdots ,N)$, $l_{N+1}=l_{m}$, ${\left(YI\right)}_{N+1}=Y_{m}I_{m}$, $m_{N+1}=M_{m}$.

(5) $$\left\{ \begin{array}{l} {\left(YI\right)}_{i}=\left\{\int_{(i+1)l_{0}}^{il_{0}}w_{b}(x)\left[Y_{b}\int_{-t_{b}+z_{N}}^{z_{N}}z^{2}dz+Y_{p}\int_{z_{N}}^{t_{p}+z_{N}}z^{2}dz\right]dx\right\}/l_{0}{,}_{}(i=1,2,3,\cdots ,N)\\ {\left(YI\right)}_{N+1}=Y_{m}I_{m}=w_{m}\left[Y_{m}\int_{-t_{m}/2}^{t_{m}/2}z^{2}dz\right]=\frac{w_{m}Y_{m}t_{m}^{3}}{12}\\ m_{i}=\int_{(i+1)l_{0}}^{il_{0}}\left(\rho_{b}t_{b}+\rho_{p}t_{p}\right)w_{b}(x)dx/l_{0}{,}_{}(i=1,2,3,\cdots ,N)\\ m_{N+1}=M_{m}=\rho_{m}w_{m}t_{m} \end{array}\right.$$

The boundary conditions of the mode function $\phi_{i}(x)$ is that (1) the displacement and rotation angle of the clamping end of each microbeam are zero, (2) the bending moment and shear force of the free end of each microbeam are zero, and (3) the displacement, rotation angle, bending moment, and shear force at the connection of each microbeam are all equal, as shown in Equation (6). According to the boundary conditions of the mode function, the *r*th order natural circular frequency of the *i*th microbeam can be solved as $\omega_{ir}=\lambda_{{}_{ir}}^{2}\sqrt{{\left(YI\right)}_{i}/\left(m_{i}l_{i}{}^{4}\right)}$, where $\lambda_{ir}$ is the characteristic value of the *r*th undamped vibration mode of the *i*th microbeam; the detailed solution process of the natural circular frequency can be seen in the in the Electronic Supplementary Information (Section S1).

(6) $$\begin{array}{cc} \left\{ \begin{array}{l} \phi_{1}\left(0\right)=0\\ {\phi_{1}^{\prime }\left(x\right)|}_{x=0}=0\\ {\phi_{N+1}^{\prime \prime }\left(x\right)|}_{x=l_{b}+l_{m}}=0\\ {\phi_{N+1}^{\prime \prime \prime }\left(x\right)|}_{x=l_{b}+l_{m}}=0 \end{array}\right. & \end{array}\left\{ \begin{array}{l} \phi_{i}\left(x_{i}\right)=\phi_{i+1}\left(x_{i}\right)\\ {\phi_{i}^{\prime }\left(x\right)|}_{x=x_{i}}={\phi_{i+1}^{\prime }\left(x\right)|}_{x=x_{i}}\\ {{\left(YI\right)}_{i}\phi_{i}^{\prime \prime }\left(x\right)|}_{x=x_{i}}={{\left(YI\right)}_{i+1}\phi_{i+1}^{\prime \prime }\left(x\right)|}_{x=x_{i}}\\ {{\left(YI\right)}_{i}\phi_{i}^{\prime \prime \prime }\left(x\right)|}_{x=x_{i}}={{\left(YI\right)}_{i+1}\phi_{i+1}^{\prime \prime \prime }\left(x\right)|}_{x=x_{i}} \end{array}\right.\begin{array}{cc} & \left(i=1,2,3,\ldots ,N\right) \end{array}$$

Then we analyze the orthogonality of the mode function and mass normalization, and obtain the orthogonality of the mode function (see Equation (7)) and the mode constant (see Equation (8)), where $\begin{array}{cc} \phi_{ir}(x)=C_{r}\begin{array}{c} \psi_{ir}(x) \end{array}, & 0\leq x\leq l_{0} \end{array}$ is the *r*th mode function of the *i*th microbeam. The detailed process of the proof of the orthogonality of the modal function can be found in the Electronic Supplementary Information (Section S2).

(7) $$\left\{ \begin{array}{l} \sum_{i=1}^{N}\left[\int_{(i-1)l_{0}}^{il_{0}}\phi_{ir}(x)m_{i}\phi_{is}(x)dx\right]+\int_{l_{b}}^{l_{b}+l_{m}}\phi_{(N+1)r}(x)M_{m}\phi_{(N+1)s}(x)dx=\delta_{rs}\\ \sum_{i=1}^{N}\left[\int_{(i-1)l_{0}}^{il_{0}}\phi_{ir}(x){\left(YI\right)}_{i}\frac{d^{4}\phi_{is}(x)}{dx^{4}}dx\right]+\int_{l_{b}}^{l_{b}+l_{m}}\phi_{(N+1)r}(x)Y_{m}I_{m}\frac{d^{4}\phi_{(N+1)s}(x)}{dx^{4}}dx=\omega_{ir}^{2}\delta_{rs} \end{array}\right.$$

(8) $$C_{r}=\sqrt{\frac{1}{\sum_{i=1}^{N}\int_{0}^{l_{0}}m_{i}\psi_{ir}^{2}(x)dx+\int_{0}^{l_{m}}M_{m}\psi_{(N+1)r}^{2}(x)dx}}$$

Thus, the bidirectional coupled DP dynamics model of the device under modal coordinates is obtained (see in the Electronic Supplementary Information (Section S3)). Finally, the vibration response, the output voltage, and the output power are derived. The details of the steady-state solution solving process can be seen in the *Electronic Supplementary Information*. To obtain the maximum electrical response, the device generally works near the basic natural frequency or a certain high-order natural frequency, that is *f* *≈ f_r_.* Therefore, the single-mode steady-state solution of the bidirectional coupled DP model is as follows.

The single-mode form of the steady-state modal mechanical response:

(9) $$\eta_{r}\left(t\right)=\frac{F_{r}\left(i2\pi fC_{p}+\frac{1}{R_{l}}\right)e^{i2\pi ft}}{\left(i2\pi fC_{p}+\frac{1}{R_{l}}\right)\left[4\pi^{2}\left(f_{r}^{2}-f^{2}+i2\zeta_{r}f_{r}f\right)\right]+i2\pi f\Theta_{r}^{2}}$$

The single-mode form of the relative displacement of the *i*th microbeam:

(10) $$z_{i}(x,t)=\frac{F_{r}\left(i2\pi fC_{p}+\frac{1}{R_{l}}\right)\phi_{ir}(x)e^{i2\pi ft}}{\left(i2\pi fC_{p}+\frac{1}{R_{l}}\right)\left[4\pi^{2}\left(f_{r}^{2}-f^{2}+i2\zeta_{r}f_{r}f\right)\right]+i2\pi f\Theta_{r}^{2}}\overset{}{}(i-1)l_{0}\leq x\leq il_{0}$$

The single-mode form of steady-state voltage response and power response on load resistance:

(11) $$\left\{ \begin{array}{l} V(t)=\frac{-i2\pi f\Theta_{r}F_{r}e^{i2\pi ft}}{\left(i2\pi fC_{p}+\frac{1}{R_{l}}\right)\left[4\pi^{2}\left(f_{r}^{2}-f^{2}+i2\zeta_{r}f_{r}f\right)\right]+i2\pi f\Theta_{r}^{2}}\\ P(t)=\frac{1}{R_{l}}{\left\{\frac{-i2\pi f\Theta_{r}F_{r}e^{i2\pi ft}}{\left(i2\pi fC_{p}+\frac{1}{R_{l}}\right)\left[4\pi^{2}\left(f_{r}^{2}-f^{2}+i2\zeta_{r}f_{r}f\right)\right]+i2\pi f\Theta_{r}^{2}}\right\}}^{2} \end{array}\right.$$

where $\Theta_{r}$ is the modal electromechanical coupling term, $F_{r}$ is the modal force function, $C_{p}$ is the internal capacitance of the piezoelectric layer, and $\zeta_{r}$ is the modal damping ratio. In this paper, the orthogonal condition is used to combine the internal damping and external damping into the modal damping ratio, and the voltage frequency response function at resonance is used to identify the damping ratio of the device in the actual test environment.

(12) $$\left\{ \begin{array}{l} \Theta_{r}=\sum_{i=1}^{N}\int_{(i-1)l_{0}}^{il_{0}}\chi \frac{d^{2}\phi_{ir}\left(x\right)}{dx^{2}}dx=-e_{31}\left(z_{N}+\frac{t_{p}}{2}\right)\left[\sum_{i=1}^{N}\int_{(i-1)l_{0}}^{il_{0}}w_{b}(x)\frac{d^{2}\phi_{ir}\left(x\right)}{dx^{2}}dx\right]\\ F_{r}=-B_{0}\Omega^{2}\left[\sum_{i=1}^{N}\int_{(i-1)l_{0}}^{il_{0}}m_{i}\phi_{ir}\left(x\right)dx+\int_{l_{b}}^{l_{b}+l_{m}}M_{m}\phi_{(N+1)r}\left(x\right)dx\right]\\ C_{p}=\frac{\varepsilon_{33}^{S}\int_{x=0}^{l_{b}}w_{b}(x)dx}{t_{p}} \end{array}\right.$$

## 3. Results and Discussion

### 3.1. Model Analysis and Verification

**Figure 2:**
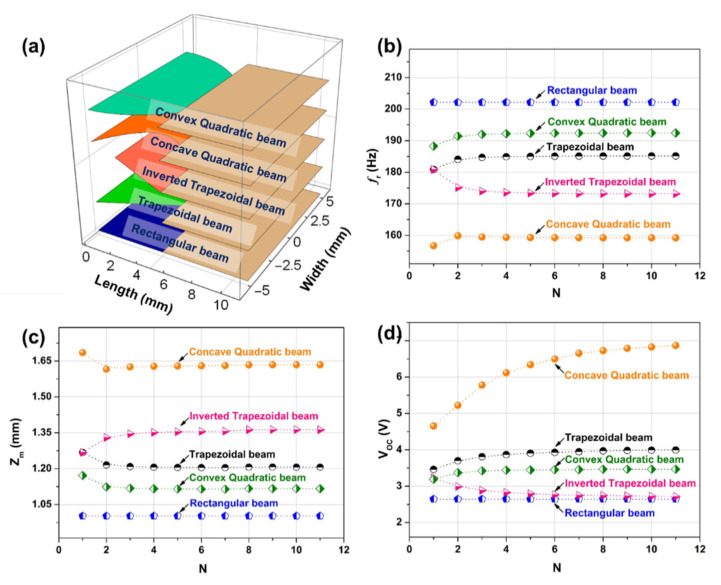
(**a**) The length and width dimension parameters of five MEMS variable cross-section cantilever beam based piezoelectric vibration energy harvesters with different cross-sectional shapes. The variations of the resonance frequency (**b**), the maximum displacement of the tip mass (**c**), and the open-circuit voltage (**d**) of the five devices with different cross-sectional shapes with the number of equal divisions N, when the external excitation acceleration is 1 g.

Five kinds of MEMS VCSCB based PVEHs with different cross-sectional shapes were selected to study the influence of the number of equal divisions (*N*) on the convergence of vibration mode and electrical response of the constructed model. Figure 2a shows the length and width dimension parameters of these five kinds of PVEHs. The beam widths of five VCSCB structures with different cross-sectional shapes are shown in Equation (13), where $w_{1}(x)$, $w_{2}(x)$, $w_{3}(x)$, $w_{4}(x)$, and $w_{5}(x)$ represent the width of the rectangular beam, the trapezoidal beam, the inverted trapezoidal beam, the concave quadratic beam, and the convex quadratic beam type, respectively. $w_{1}$ and $w_{2}$ are the width parameters of the bound end of the beam and the joint between the beam and the tip mass, respectively. The material parameters of the beam and piezoelectric materials in this paper are shown in Table S1.

(13) $$\left\{ \begin{array}{l} w_{1}(x)=w_{1}\\ w_{2}(x)=w_{1}-\frac{w_{1}-w_{2}}{l_{b}}x\\ w_{3}(x)=w_{2}+\frac{w_{1}-w_{2}}{l_{b}}x\\ w_{4}(x)=w_{1}-\frac{w_{1}+w_{2}}{l_{b}}x+2\frac{w_{2}}{l_{b}{}^{2}}x^{2}\\ w_{5}(x)=w_{1}+\frac{5w_{2}-3w_{1}}{l_{b}}x+\frac{2w_{1}-4w_{2}}{l_{b}{}^{2}}x^{2} \end{array}\right.$$

Since the thickness of the electrode is much smaller than the thickness of the piezoelectric layer and the substrate layer, it can be ignored in the analysis process. The thicknesses of the Si substrate layer, the AlN piezoelectric layer, and the tip mass block were selected as 50, 1, and 500 μm, respectively, in the analysis process. The widths of the clamping ends of the five devices are 10 mm, and the width of the joint between the beam and the tip mass is 6 mm. Figure 2b–d respectively show the variations of the resonance frequency ($f_{r}$), the maximum displacement of the tip mass ($z_{m}$), and the open-circuit voltage ($V_{oc}$) of the five devices with different cross-sectional shapes with the number of equal divisions at the excitation acceleration of 1 g. It can be seen that the performance parameters of the rectangular beam based device do not change with *N*. The $f_{r}$ and $z_{m}$ of the other four devices gradually tend to be constant with the increase of N, and the convergence speed is faster. For the $V_{oc}$, the concave quadratic beam converges slowly to a constant value, while other beams can quickly converge to a constant value. Then, we take the above-mentioned trapezoidal cantilever beam device as an example, and use the ANSYS finite element model (FEM) to verify the constructed bidirectional coupled DP model. The relevant settings and attributes in the ANSYS modeling process can be seen in the Electronic Supplementary Information (Section S4). The comparison of the modal and harmonic response results of the trapezoidal beam PVEH obtained from the ANSYS FEM and the constructed bidirectional coupled DP model is presented in Table 1. We can see from the table that the resonance frequency, the maximum displacement of the tip mass, the open-circuit voltage, and the maximum load output power ($P_{\max}$) of the ANSYS FEM and the constructed bidirectional coupled DP model under the condition of the same size and structural parameters are in good agreement with a percentage error of less than 4.96%. Therefore, the constructed theoretical model can be used to guide optimal design and predict the electrical output.

In short, the constructed bidirectional coupled DP model not only considers the influence of the mechanical part on the electrical part, that is, the positive piezoelectric effect, but also considers the influence of the electrical part on the mechanical part, that is, the inverse piezoelectric effect. It can effectively solve the problem of poor prediction accuracy of the unidirectional coupled distributed parameter model under strong electromechanical coupling. The result shows that the proposed analytical model has the advantages of high prediction accuracy, good convergence, fast calculation speed, analytical solution available, and suitable for variable cross-section beam structures of any shape in d_31_ mode. Thus, the analytical model can provide a fast and effective theoretical basis for the design and optimization of the MEMS VCSCB based PVEHs.

### 3.2. Influence of the VCSCB’s Shape on the Device’s Output

**Figure 3:**
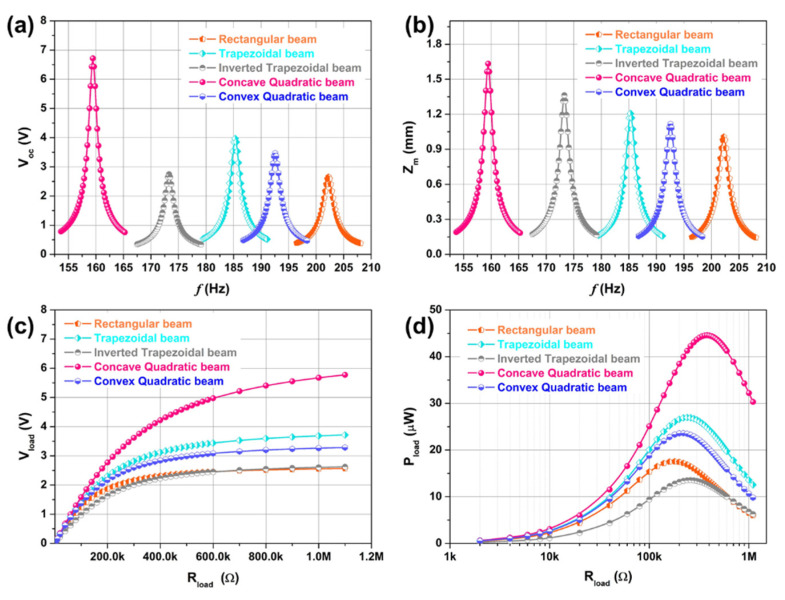
Output performance of five MEMS variable cross-section cantilever beam based piezoelectric vibration energy harvesters with different cross-sectional shapes. (**a**) frequency response curve of open-circuit voltage, (**b**) frequency response curve of the maximum displacement of the tip mass, (**c**) load characteristic curve of output voltage, and (**d**) load characteristic curve of output power. These cases show results for an external excitation acceleration of 1 g.

Based on the constructed bidirectional coupled DP model, we analyze the influence of the shape of the MEMS VCSCB on the output performance of the device. Figure 3 shows the output performance of five MEMS variable cross-section cantilever beam based piezoelectric vibration energy harvesters with different cross-sectional shapes. Figure 3a–d represents the frequency response curve of open-circuit voltage, the frequency response curve of the maximum displacement of the tip mass, the load characteristic curve of output voltage, and the load characteristic curve of output power, respectively. It can be seen that the open-circuit voltage ($V_{oc}$), the load voltage ($V_{load}$), and the load power ($P_{load}$) of the trapezoidal beam, the concave quadratic beam, and the convex quadratic beam are better than that of the rectangular beam when the beam width is inconsistent and other structural parameters are the same. Further, the output performance of the concave quadratic beam is the best, the $f_{r}$ of the concave quadratic beam is the lowest, but the $z_{m}$, $V_{oc}$, $V_{load}$, and $P_{load}$ are the largest. It is because that the trapezoidal beam, the concave quadratic beam, and the convex quadratic beam have a more uniform axial stress distribution than the rectangular beam, resulting in greater total axial strain energy. It can also be seen from the figure that although the inverted trapezoidal beam has a lower $f_{r}$ and a greater $z_{m}$ than the rectangular beam, their $V_{oc}$ and $V_{load}$ are basically the same, and the $P_{load}$ of the inverted trapezoidal beam is lower. It is because the size of the inverted trapezoidal beam’s bound end with the largest axial stress is smaller than that of the rectangular beam, and the total strain energy will be reduced. In general, we can use the method of optimizing the shape of the MEMS VCSCB to optimize the axial stress distribution of the piezoelectric layer, to reduce the resonant frequency of the device, and to improve the electrical output performance of the device.

### 3.3. Influence of the VCSCB’s Structural Parameters on the Device’s Output

Based on the constructed DP dynamics model, and taking the MEMS trapezoidal cantilever beam (TCB) based PVEH as an example, the influence of the main structural parameters on the performance of the device is analyzed. The example device is mainly composed of the Si substrate layer, the SiO_2_ oxide layer, the Pt bottom electrode layer, the AlN piezoelectric layer, the Al top electrode layer, and the tip mass; the corresponding thicknesses are $t_{b}$, $t_{SiO_{2}}$, $t_{Pt}$, $t_{p}$, $t_{Al}$, and $t_{m}$, respectively. The sum of $l_{b}$ and $l_{m}$ is 10 mm, the widths of the clamping end are ($w_{1}$) the tip mass ($w_{m}$) are both 8 mm, and the width of the joint between the beam and the tip mass is $w_{2}$. Considering the processing technology, the main structural parameters that affect the performance of the TCB based PVEH are $l_{b}$, $w_{2}$, and $t_{b}$. During analysis, $t_{SiO_{2}}$, $t_{Pt}$, $t_{p}$, $t_{Al}$, and $t_{m}$ are set to 300 nm, 120 nm, 1 μm, 900 nm, and 400 μm, respectively. The external excitation acceleration is set to 0.5g.

**Figure 4:**
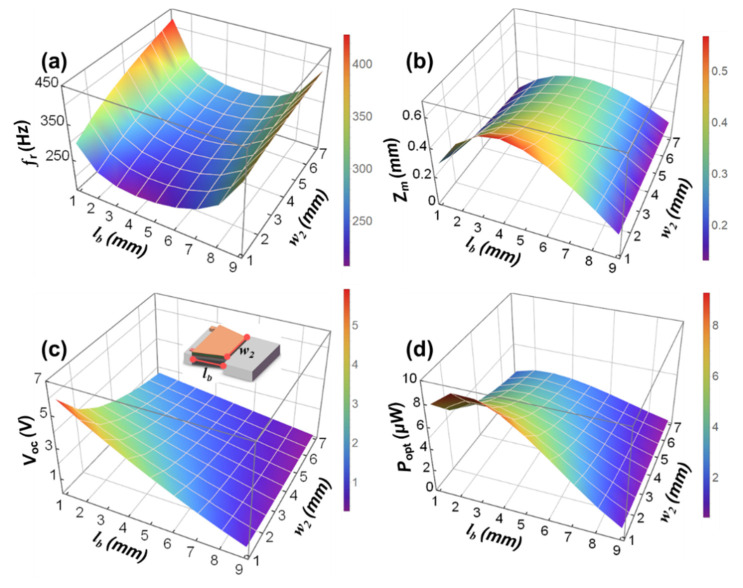
Variations of the resonance frequency $f_{r}$ (**a**), the maximum displacement of the tip mass $z_{m}$ (**b**), the open-circuit voltage $V_{oc}$ (**c**), and the optimal load output power $P_{opt}$ (**d**) of the MEMS TCB based PVEH under different $l_{b}$ and $w_{2}$.

Figure 4a–d respectively show the variations of the resonance frequency ($f_{r}$), the maximum displacement of the tip mass ($z_{m}$), the open-circuit voltage ($V_{oc}$), and the optimal load output power ($P_{opt}$) of the MEMS TCB based PVEH under different $l_{b}$ and $w_{2}$. At this time, the beam thickness $t_{b}$ is set to 50 μm. Figure S1a–d are the corresponding contour maps. It can be seen that while keeping other parameters constant, the $f_{r}$ of the device first decreases and then increases as the $l_{b}$ increases, the $V_{oc}$ gradually decreases as the $l_{b}$ increases, and both the $P_{opt}$ and the $z_{m}$ increase first and then decrease with the increase of $l_{b}$. It can be seen that when the beam length is between 2 and 6 mm, the resonant frequency changes slowly, and there is a minimum value. At the same time, the output power reaches the maximum when the beam length is about 2.5 mm. However, the displacement of the end of the mass is relatively large between 2 and 6 mm, and there is a maximum value. Therefore, when selecting the length of the silicon cantilever beam according to this rule in the structural design, in order to ensure that the resonant frequency of the device matches the environmental frequency, to ensure high output power and small mass end displacement, while taking into account the appropriate output voltage and stress, the beam length should be selected between 2 and 6 mm close to 2 mm.

It can also be seen that while keeping other parameters constant, within a certain range, as $w_{2}$ gradually decreases from 7 to 1 mm, the $f_{r}$ of the device gradually decreases, and the $z_{m}$, $V_{oc}$, and $P_{opt}$ all increase with the decrease of $w_{2}$. Thus, the smaller the $w_{2}$, the smaller the bending stiffness of the TCB. The smaller the $w_{2}$, the smaller the resonance frequency of the TCB. The smaller the $w_{2}$, the greater the displacement of the tip mass. The smaller the $w_{2}$, the greater the strain energy per unit area of the piezoelectric layer. In short, under the condition of ensuring that the resonant frequency of the device matches the environmental frequency and the mass end displacement is moderate, reducing the width of the joint between the beam and the tip mass of a variable cross-section beam device can effectively improve the output performance of the device.

**Figure 5:**
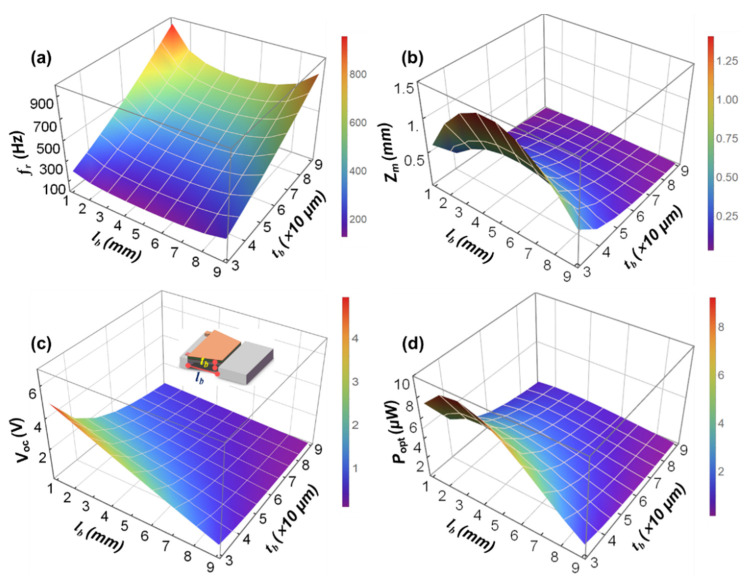
Variations of the resonance frequency $f_{r}$ (**a**), the maximum displacement of the tip mass $z_{m}$ (**b**), the open-circuit voltage $V_{oc}$ (**c**), and the optimal load output power $P_{opt}$ (**d**) of the MEMS TCB based PVEH under different $l_{b}$ and $t_{b}$.

Figure 5a–d respectively show the variations of the $f_{r}$, the $z_{m}$, the $V_{oc}$, and the $P_{opt}$ of the MEMS TCB based PVEH under different $l_{b}$ and $t_{b}$; Figure S2a–d shows the corresponding contour maps. At this time, $w_{2}$ is set to 6 mm. It can be seen that while keeping other parameters constant, within a certain range, as the thickness of the Si cantilever increases, the bending stiffness of the TCB increases, and the strain energy per unit area in the piezoelectric layer decreases, so that the $f_{r}$ of the device gradually increases, and the $z_{m}$, the $V_{oc}$, and the $P_{opt}$ all gradually decrease. At the same time, the larger the beam thickness, the slower the $z_{m}$, the $V_{oc}$, and the $P_{opt}$, and they decrease. The thickness of the cantilever beam has a greater impact on performance parameters than the length of the cantilever beam on performance parameters. Under the conditions of ensuring that the resonant frequency of the device matches the environmental frequency, the mass end displacement is moderate, and the substrate layer stress and the process conditions meet the requirements, reducing the thickness of the variable cross-section beam can effectively improve the output performance of the device.

In short, $l_{b}$, $w_{2}$, and $t_{b}$ can effectively adjust the resonance frequency and electrical output performance of the MEMS TCB based PVEH.

### 3.4. Prototype Processing and Performance Analysis

**Figure 6:**
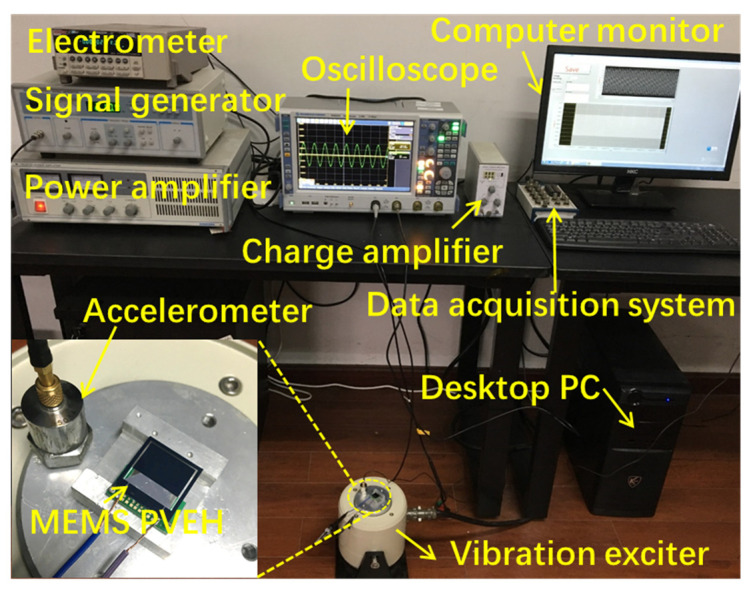
Digital photograph of the experimental setup of testing the device.

To verify the prediction accuracy of the constructed DP dynamic model, we developed an AlN MEMS TCB based PVEH using standard MEMS technology. The specific process flow has ten steps, mainly including the preparation and patterning of the AlN piezoelectric film, the preparation and patterning of the top and bottom electrodes, and the preparation of the cantilever beams. The detailed processing process of the device can be found in Table S2 in the Electronic Supplementary Information. The specific structural parameters are that the $l_{b}$ and the $l_{m}$ are 4 and 9 mm. The $t_{b}$, $t_{SiO_{2}}$, $t_{Pt}$, $t_{p}$, $t_{Al}$, and $t_{m}$ are 50 μm, 200 nm, 120 nm, 1 μm, 900 nm, and 400 μm, respectively. The $w_{1}$, $w_{2}$, and $w_{m}$ are 14, 9, and 14 mm, respectively, and the external excitation acceleration is 1 g. The experimental setup can be found in the Figure 6 in the revised manuscript. The MEMS VCSCB based PVEH was driven by a vibration exciter which was controlled by a function signal generator and a power amplifier. The output signals of the device were measured by the electrometer and the oscilloscope. The vibration acceleration was measured by an accelerometer mounted on the vibration exciter, and the acceleration signal was transmitted by a charge amplifier and data acquisition system to a desktop PC which displayed it.

**Figure 7:**
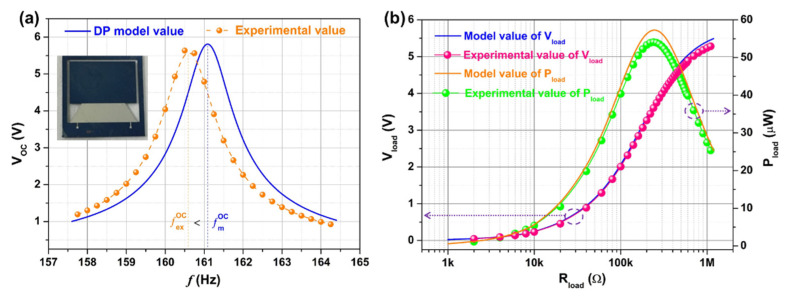
The open-circuit voltage frequency response curve (**a**) and the load output characteristic curve (**b**) of the MEMS TCB based PVEH prototype at the external excitation acceleration of 1 g.

Figure 7a is the open-circuit voltage frequency response curve of the device. It can be seen that the experimental values of the $f_{r}$ and the $V_{oc}$ of the device are 160.6 Hz and 5.64 V, respectively. The theoretical values of the $f_{r}$ and the $V_{oc}$ of the device are 161.1 Hz and 5.83 V, respectively. The experimental values of the $f_{r}$ and the $V_{oc}$ are slightly lower than the theoretical values. The main reason may be the slight deviation between the thickness of each layer of the film and the design value during the device processing. Figure 7b is the load output characteristic curve of the device. It can be seen from the curve that as the load resistance increases, the theoretical and experimental values of the load output voltage of the device both gradually increase and then become stable. Under a load resistance of 1 MΩ, the theoretical and experimental values of the device’s load voltage are 5.4 and 5.24 V, respectively. It can be seen from the curve that as the load resistance increases, the theoretical value and the experimental value of the load output power of the device both increase first and then decrease, and both have a maximum value. The experimental value of the peak output power and normalized power density of the device are 54.1 μW and 742 μW/cm^3^/g^2^, respectively, under the optimized load of 240 kΩ. The theoretical value of the peak output power and normalized power density of the device are 57.2 μW and 784 μW/cm^3^/g^2^, respectively, under the optimized load of 260 kΩ. In short, the experimental and theoretical values of the load voltage and load power of the device are basically consistent. It shows that the constructed theoretical model has effective prediction accuracy. Figure S3 shows the electrical output comparison between the trapezoidal beam based PVEH prototype and the rectangular beam based PVEH prototype in the open circuit condition and the external load condition. It can be seen from Figure S3a that the open circuit voltage of the trapezoidal beam device (5.64 V) is greater than that of the rectangular beam device (3.95 V), and the open circuit resonance frequency of the trapezoidal beam device is lower than that of the rectangular beam device. Figure S3b shows the output power of the trapezoidal beam based PVEH and the rectangular beam based PVEH under different load resistances. As can be seen from the figure, as the load resistance increases, the output power first increases and then decreases, and both have a maximum value. The trapezoidal beam device achieves a maximum value of 54.1 μW at 240 KΩ, and the rectangular beam device achieves a maximum value of 38.6 μW at 180 KΩ. The maximum output power of the trapezoidal beam PVEH is significantly higher than that of the rectangular beam PVEH. The experimental values of open-circuit voltage and peak load output power the MEMS TCB based PVEH prototype are respectively 1.43 and 1.4 times those of the rectangular beam prototype under the same experimental conditions. The experimental results verify that the trapezoidal beam PVEH has better electrical output performance than the rectangular beam PVEH when only the beam width at the junction of the cantilever beam and the mass is different, and other structural parameters are the same.

**Figure 8:**
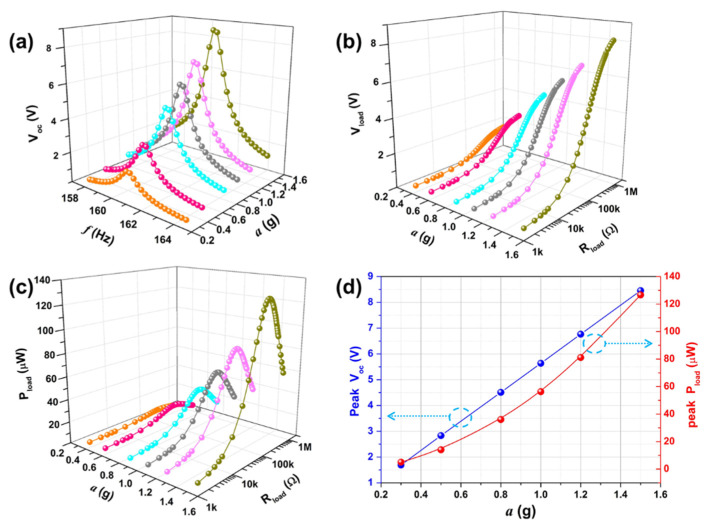
The output performance of the MEMS TCB based PVEH prototype under different accelerations, (**a**) open-circuit voltage of the device under different excitation frequencies and excitation accelerations, (**b**) load voltage of the device under different excitation acceleration and load resistance, (**c**) load power of the device under different excitation acceleration and load resistance, (**d**) variations of the peak open-circuit voltage and the peak load power of the device under different excitation acceleration.

Figure 8 shows the output performance of the MEMS TCB based PVEH prototype under different accelerations. It can be seen from Figure 8a–c that as the excitation acceleration increases, the open-circuit voltage, the load voltage, and the load power of the device all continue to increase. Under the specified acceleration excitation, the load voltage first increases with the increase of load resistance and then gradually stabilizes. The load power first increases and then decreases with the increase of the load resistance, and reaches the peak value when the load resistance is equal to the internal resistance. It can be seen from Figure 8d that the peak value of the open-circuit voltage of the device has a linear relationship with the excitation acceleration, and the peak value of the load power of the device has a quadratic relationship with the excitation acceleration. Under the excitation of 1 g acceleration, the load voltage and load power can reach 3.68 V and 54.1 μW at the load resistance of 240 KΩ, respectively. The normalized power density of the device can reach 784 μW/cm^3^/g^2^. In view of the high normalized output power density of the device, the device has wide application prospects in the power supply of the wireless sensor network node. For the energy supply of intermittent, low-transmission frequency wireless sensor network nodes, the device or the energy supply array based on the device in this paper have potential application prospects. For example, the device can be used to power the machine’s fault monitoring node. The schematic diagram of the fault monitoring node is shown in Figure S4. In this node, the output signal of the vibration energy harvester is conditioned by a power management circuit to supply power to the acceleration sensor and the microcontroller, and excess energy is stored in the thin film battery. Once the acceleration sensor receives power, it begins to monitor the vibration signal and sends it to the relay node, after processing by the microcontroller.

## 4. Conclusions

In this work, we construct, analytically solve, and verify the bidirectional coupled distributed parameter dynamics theoretical model of the MEMS VCSCB based PVEH, and the mapping relationship and influence law of the structural and material parameters of the device on its normalized output power density are obtained, which laid an important theoretical foundation for structural design and optimization, performance improvement, and output prediction of the MEMS PVEH. Based on the constructed model, the output of five kinds of MEMS VCSCB based PVEHs with different cross-sectional shapes were compared and analyzed. The results show that the VCSCB based PVEH with a concave quadratic beam shape has the best output due to the uniform surface stress distribution. Additionally, the influence of the main structural parameters of the MEMS TCB based PVEH on the output performance of the device is theoretically analyzed. Finally, a prototype of the AlN TCB based PVEH is developed. The peak open-circuit voltage and normalized power density of the device can reach 5.64 V and 742 μW/cm^3^/g^2^, which is in good agreement with the theoretical model value. The prototype has wide application prospects in the power supply of the wireless sensor network node such as the structural health monitoring system and the Internet of Things.

## Figures and Tables

**Table 1:** Output performance comparisons of the trapezoidal beam PVEH by using the ANSYS FEM and the constructed bidirectional coupled DP model.

	ANSYS FEM	CDP Model	% Error
*f_r_* (Hz)	185.26	190.15	2.64%
*Z_m_* (mm)	1.21	1.27	4.96%
*V_oc_* (V)	3.98	4.15	4.27%
*P_max_* (μW)	27.11	28.06	3.50%

## Supplementary Materials

The following are available online at https://www.mdpi.com/article/10.3390/mi12070772/s1, Section S1: Solving process of the undamped natural circular frequency, Section S2: The proof of the orthogonality of the modal function, Section S3: Bidirectionally coupled distributed parameter model and its steady-state solution under modal coordinates, Section S4: The relevant settings and attributes in the ANSYS modeling process, Table S1: Material properties of the beam and piezoelectric materials, Table S2: Process flow of the MEMS trapezoidal cantilever beam based PVEH, Figure S1: Contour maps of variations of the resonance frequency, the maximum displacement of the tip mass, the open-circuit voltage, and the optimal load output power of the MEMS TCB based PVEH under different $l_{b}$ and $w_{2}$, Figure S2: Contour maps of variations of the resonance frequency, the maximum displacement of the tip mass, the open-circuit voltage, and the optimal load output power of the MEMS TCB based PVEH under different $l_{b}$ and $t_{b}$, Figure S3: The open-circuit voltage frequency response curve and the load output power curve of the MEMS trapezoidal and rectangular beam based PVEH prototypes at the external excitation acceleration of 1 g, Figure S4: Schematic diagram of fault monitoring node based on the MEMS PVEH.
