# Supplementary material for: Theoretical and Experimental Studies on MEMS Variable Cross-Section Cantilever Beam Based Piezoelectric Vibration Energy Harvester
Source: Micromachines (Basel). 2021 Jun 30;12(7):772. doi: 10.3390/mi12070772 (PMC8304101; doi:10.3390/mi12070772)
Supplement: Supplementary file 1 [file micromachines-12-00772-s001.zip › micromachines-1254395-supplementary.pdf]

Electronic Supplementary Information

Theoretical and Experimental Studies on MEMS Variable Cross-Section Cantilever Beam based Piezoelectric Vibration Energy Harvester

Xianming He [1,2,*], Dongxiao Li [1,2], Hong Zhou [1,2], Xindan Hui [1,2] and Xiaojing Mu [1,2,*]

[1] Key Laboratory of Optoelectronic Technology & Systems, Ministry of Education, Chongqing University, Chongqing 400044, P.R. China; lidongxiao@cqu.edu.cn (D.L.); zhlf@cqu.edu.cn (H.Z.); huixindan@cqu.edu.cn (X.H.)

[2] International R & D center of Micro-nano Systems and New Materials Technology, Chongqing University, Chongqing 400044, P.R. China

* Correspondence: hexianming@cqu.edu.cn (X.H.); mxjacj@cqu.edu.cn (X.M.)

Section S1. Solving process of the undamped natural circular frequency

The undamped natural frequencies and mode shapes are applicable to proportionally damped systems, so we can analyze the damped system by solving the undamped system. The undamped governing equation of free vibrations for the MEMS variable cross-section cantilever beam is given as follows.

$$\frac{\partial^2}{\partial x^2}\left[YI(x)\frac{\partial^2 z(x,t)}{\partial x^2}\right]+m(x)\frac{\partial^2 z(x,t)}{\partial t^2}=0 \tag{S1}$$

The method of separation of variables can be used to solve Equations (S1) by separating the spatial and temporal functions as $z(x,t)=\phi(x)\eta(t)$. Based on the infinitesimal method, the undamped free vibration equation of each microbeam can be obtained after the variables are separated.

$$\frac{(YI)_i}{m_i}\frac{1}{\phi_i(x)}\frac{d^4\phi_i(x)}{dx^4}=-\frac{1}{\eta_i(t)}\frac{d^2\eta_i(t)}{dt^2} \tag{S2}$$

The left-hand side of Equation (S2) depends on x alone, while the right-hand side depends on t alone. Since x and t are independent variables, the standard argument in the method of separation of variables states that both sides of Equation (S2) must be equal to the same constant ω_i^2. So,

$$\frac{d^4\phi_i(x)}{dx^4}-\omega_i^2\frac{m_i}{(YI)_i}\phi_i(x)=0\,,\quad \frac{d^2\eta_i(t)}{dt^2}+\omega^2\eta_i(t)=0 \tag{S3}$$

The solution of Equation (S3) is as follows.

$$\begin{cases}\phi_i(x)=c_{1i}\cos\left(\frac{\lambda_i}{l_i}x\right)+c_{2i}\sin\left(\frac{\lambda_i}{l_i}x\right)+c_{3i}\cosh\left(\frac{\lambda_i}{l_i}x\right)+c_{4i}\sinh\left(\frac{\lambda_i}{l_i}x\right)\\ \eta_i(t)=H_i e^{i\omega_i t}\end{cases} \tag{S4}$$

Where $c_{ki}(k=1,\ 2,\ 3,\ 4)$ and H_i are unknown constants, λ_i and ω_i are the eigenvalue and eigenfrequency (natural circular frequency) of the ith undamped vibration mode, respectively. So the eigenfrequency is $\omega_i=\lambda_i^2\sqrt{\frac{(YI)_i}{m_i l_i^4}}$. Based on the idea of the infinitesimal method, the vibration response $z(x,t)$ of the MEMS variable cross-section cantilever beam based piezoelectric vibration energy harvester can be expressed as a piecewise function of the vibration response of the N+1 segment rectangular beam. The vibration response of the ith-segment microbeam can be defined as $z_i(x,t)=\sum_{r=1}^{\infty}\phi_{ir}(x)\eta_{ir}(t)\quad x_{i-1}\le x\le x_i$.

Where $x_i=il_0,\ (i=1,\ 2,\ 3,\ ...\ N)$ and $x_{N+1}=l_b+l_m$. Therefore, the modal shape function and the rth order modal shape function of the vibration response of the ith-segment microbeam can be written as follows, respectively. $A_{ki}\ (k=1,2,3,4;\ i=1,2,3,\cdots,N+1)$ is a constant.

$$\begin{cases}\phi_i(x)=A_{1i}\cos\left(\frac{\lambda_i}{l_i}x\right)+A_{2i}\sin\left(\frac{\lambda_i}{l_i}x\right)+A_{3i}\cosh\left(\frac{\lambda_i}{l_i}x\right)+A_{4i}\sinh\left(\frac{\lambda_i}{l_i}x\right) & x_{i-1}\le x\le x_i\\ \phi_{ir}(x)=A_{1i}\cos\left(\frac{\lambda_{ir}}{l_i}x\right)+A_{2i}\sin\left(\frac{\lambda_{ir}}{l_i}x\right)+A_{3i}\cosh\left(\frac{\lambda_{ir}}{l_i}x\right)+A_{4i}\sinh\left(\frac{\lambda_{ir}}{l_i}x\right) & x_{i-1}\le x\le x_i\end{cases} \tag{S5}$$

Now the resonant circular frequency can be expressed as follows.

$$\omega_n=\omega_{ni}=\lambda_i^2\sqrt{\frac{(YI)_i}{m_i l_i^4}}\quad (i=1,2,3,\cdots,N+1) \tag{S6}$$

In Equation (S6), $(YI)_i$ and m_i respectively satisfy the following equations.

$$\begin{cases}(YI)_i=\left\{\int_{(i+1)l_0}^{il_0} w_b(x)\left[Y_b\int_{-t_b+z_N}^{z_N} z^2dz+Y_p\int_{z_N}^{t_p+z_N} z^2dz\right]dx\right\}/l_0,\ (i=1,2,3,\cdots,N)\\ (YI)_{N+1}=Y_mI_m=w_m\left[Y_m\int_{-t_m/2}^{t_m/2} z^2dz\right]=\frac{w_mY_mt_m^3}{12}\\ m_i=\int_{(i+1)l_0}^{il_0}\left(\rho_bt_b+\rho_pt_p\right)w_b(x)dx/l_0,\ (i=1,2,3,\cdots,N)\\ m_{N+1}=M_m=\rho_mw_mt_m\end{cases} \tag{S7}$$

The boundary condition of the mode function $\phi_i(x)$ is that 1) the displacement and rotation angle of the clamping end of each microbeam are zero, 2) the bending moment and shear force of the free end of each microbeam are zero, and 3) the displacement, rotation angle, bending moment, and shear force at the connection of each microbeam are all equal, as shown in Equation (S8).

$$\begin{cases}\phi_1(0)=0\\ \phi_1'(x)\big|_{x=0}=0\\ \phi_{N+1}''(x)\big|_{x=l_b+l_m}=0\\ \phi_{N+1}'''(x)\big|_{x=l_b+l_m}=0\end{cases}\quad \begin{cases}\phi_i(x_i)=\phi_{i+1}(x_i)\\ \phi_i'(x)\big|_{x=x_i}=\phi_{i+1}'(x)\big|_{x=x_i}\\ (YI)_i\phi_i''(x)\big|_{x=x_i}=(YI)_{i+1}\phi_{i+1}''(x)\big|_{x=x_i}\\ (YI)_i\phi_i'''(x)\big|_{x=x_i}=(YI)_{i+1}\phi_{i+1}'''(x)\big|_{x=x_i}\end{cases}\quad (i=1,2,3,\ldots,N) \tag{S8}$$

After the coordinate translation transformation is performed on the mode function $\phi_i(x)$ in Equation (S5), Equation (S9) can be obtained.

$$\begin{cases}\phi_1(x)=\varphi_1(x)\\ \phi_i(x)=\varphi_i\left[x'+x_{i-1}\right]\quad 0\le x'\le l_i,\ (i=2,3,\cdots,N+1)\\ \varphi_i(x)=A_{1i}\cos\left(\frac{\lambda_i}{l_i}x\right)+A_{2i}\sin\left(\frac{\lambda_i}{l_i}x\right)+A_{3i}\cosh\left(\frac{\lambda_i}{l_i}x\right)+A_{4i}\sinh\left(\frac{\lambda_i}{l_i}x\right)\quad 0\le x\le l_i\end{cases} \tag{S9}$$

After the coordinate translation transformation, the boundary conditions of the mode shape function become as follows.

$$\begin{cases} \varphi_1(0)=0 \\ \varphi_1'(x)\big|_{x=0}=0 \\ \varphi_{N+1}''(x)\big|_{x=l_m}=0 \\ \varphi_{N+1}'''(x)\big|_{x=l_m}=0 \end{cases} \quad \begin{cases} \varphi_i(l_i)=\varphi_{i+1}(0) \\ \varphi_i'(x)\big|_{x=l_i}=\varphi_{i+1}'(x)\big|_{x=0} \\ (YI)_i\varphi_i''(x)\big|_{x=l_i}=(YI)_{i+1}\varphi_{i+1}''(x)\big|_{x=0} \\ (YI)_i\varphi_i'''(x)\big|_{x=l_i}=(YI)_{i+1}\varphi_{i+1}'''(x)\big|_{x=0} \end{cases} \quad (i=1,2,3,\dots,N) \tag{S10}$$

Let functions $P_i(x)$, $Q_i(x)$, $R_i(x)$, and $S_i(x)$ $(i=1,2,3,\cdots,N+1)$ satisfy the following equation.

$$\begin{cases} P_i(x)=\frac{1}{2}\left[\cosh\left(\frac{\lambda_i}{l_i}x\right)+\cos\left(\frac{\lambda_i}{l_i}x\right)\right], \quad Q_i(x)=\frac{1}{2}\left[\sinh\left(\frac{\lambda_i}{l_i}x\right)+\sin\left(\frac{\lambda_i}{l_i}x\right)\right] \\ R_i(x)=\frac{1}{2}\left[\cosh\left(\frac{\lambda_i}{l_i}x\right)-\cos\left(\frac{\lambda_i}{l_i}x\right)\right], \quad S_i(x)=\frac{1}{2}\left[\sinh\left(\frac{\lambda_i}{l_i}x\right)-\sin\left(\frac{\lambda_i}{l_i}x\right)\right] \end{cases} \tag{S11}$$

Therefore, the mode function $\varphi_i(x)$ after coordinate translation transformation can be expressed as follows.

$$\varphi_i(x)=(A_{1i}+A_{3i})P_i(x)+(A_{2i}+A_{4i})Q_i(x)+(A_{3i}-A_{1i})R_i(x)+(A_{4i}-A_{2i})S_i(x) \quad 0\le x\le l_i \tag{S12}$$

Combining Equation (S10) and the transformed mode function, the boundary condition equations shown in Equation (S13) can be obtained.

$$\begin{cases} A_{11}+A_{31}=0 \\ \frac{\lambda_1}{l_0}(A_{21}+A_{41})=0 \end{cases}$$

$$\begin{cases} (A_{1i}+A_{3i})P_i(l_i)+(A_{2i}+A_{4i})Q_i(l_i)+(A_{3i}-A_{1i})R_i(l_i)+(A_{4i}-A_{2i})S_i(l_i)=A_{1(i+1)}+A_{3(i+1)} \\ (A_{1i}+A_{3i})S_i(l_i)+(A_{2i}+A_{4i})P_i(l_i)+(A_{3i}-A_{1i})Q_i(l_i)+(A_{4i}-A_{2i})R_i(l_i)=\frac{\lambda_{i+1}}{\lambda_i}\left[A_{2(i+1)}+A_{4(i+1)}\right] \\ (A_{1i}+A_{3i})R_i(l_i)+(A_{2i}+A_{4i})S_i(l_i)+(A_{3i}-A_{1i})P_i(l_i)+(A_{4i}-A_{2i})Q_i(l_i)=\frac{(YI)_{i+1}}{(YI)_i}\left(\frac{\lambda_{i+1}}{\lambda_i}\right)^2\left[-A_{1(i+1)}+A_{3(i+1)}\right] \quad (i=1,2,3,\cdots,N) \\ (A_{1i}+A_{3i})Q_i(l_i)+(A_{2i}+A_{4i})R_i(l_i)+(A_{3i}-A_{1i})S_i(l_i)+(A_{4i}-A_{2i})P_i(l_i)=\frac{(YI)_{i+1}}{(YI)_i}\left(\frac{\lambda_{i+1}}{\lambda_i}\right)^3\left[-A_{2(i+1)}+A_{4(i+1)}\right] \end{cases} \tag{S13}$$

$$\begin{cases} \left[A_{1(N+1)}+A_{3(N+1)}\right]R_{N+1}(l_m)+\left[A_{2(N+1)}+A_{4(N+1)}\right]S_{N+1}(l_m)+\left[-A_{1(N+1)}+A_{3(N+1)}\right]P_{N+1}(l_m)+\left[-A_{2(N+1)}+A_{4(N+1)}\right]Q_{N+1}(l_m)=0 \\ \left[A_{1(N+1)}+A_{3(N+1)}\right]Q_{N+1}(l_m)+\left[A_{2(N+1)}+A_{4(N+1)}\right]R_{N+1}(l_m)+\left[-A_{1(N+1)}+A_{3(N+1)}\right]S_{N+1}(l_m)+\left[-A_{2(N+1)}+A_{4(N+1)}\right]P_{N+1}(l_m)=0 \end{cases}$$

Solving Equation (S13) can get the following formula.

$$[\mathbf{J_{N+1}}][\mathbf{J_N}][\mathbf{J_{N-1}}]\cdots[\mathbf{J_i}]\cdots[\mathbf{J_2}][\mathbf{J_1}]\begin{bmatrix}-2A_{11}\\-2A_{21}\end{bmatrix}=\begin{bmatrix}a_{11}&a_{12}\\a_{21}&a_{22}\end{bmatrix}\begin{bmatrix}-2A_{11}\\-2A_{21}\end{bmatrix}=\begin{bmatrix}0\\0\end{bmatrix} \tag{S14}$$

Assuming $(YI)_i/(YI)_{i+1}=B_i$, $\lambda_i l_{i+1}/\lambda_{i+1}l_i=\varpi_i$, Therefore, $[\mathbf{J_i}]$, $(i=1,2,3,\cdots,N)$ can be expressed as follows.

$$\begin{cases} [\mathbf{J_{N+1}}] = \begin{bmatrix} R_{N+1}(l_m) & S_{N+1}(l_m) & P_{N+1}(l_m) & Q_{N+1}(l_m) \\ Q_{N+1}(l_m) & R_{N+1}(l_m) & S_{N+1}(l_m) & P_{N+1}(l_m) \end{bmatrix} \\ [\mathbf{J_1}] = \begin{bmatrix} R_1(l_1) & \varpi_1 Q_1(l_1) & B_1\varpi_1^2 P_1(l_1) & B_1\varpi_1^3 S_1(l_1) \\ S_1(l_1) & \varpi_1 R_1(l_1) & B_1\varpi_1^2 Q_1(l_1) & B_1\varpi_1^3 P_1(l_1) \end{bmatrix}^T \\ [\mathbf{J_i}] = \begin{bmatrix} P_i(l_i) & Q_i(l_i) & R_i(l_i) & S_i(l_i) \\ \varpi_i S_i(l_i) & \varpi_i P_i(l_i) & \varpi_i Q_i(l_i) & \varpi_i R_i(l_i) \\ B_i\varpi_i^2 R_i(l_i) & B_i\varpi_i^2 S_i(l_i) & B_i\varpi_i^2 P_i(l_i) & B_i\varpi_i^2 Q_i(l_i) \\ B_i\varpi_i^3 Q_i(l_i) & B_i\varpi_i^3 R_i(l_i) & B_i\varpi_i^3 S_i(l_i) & B_i\varpi_i^3 P_i(l_i) \end{bmatrix}, \ (i = 2, 3, \cdots, N) \end{cases} \tag{S15}$$

If A_{11} and A_{21} have non-zero solutions, the determinant of the coefficient matrix of formula (S14) must be zero, so the resonance frequency equation can be obtained as follows.

$$f(\lambda_i) = \left| [\mathbf{J_m}][\mathbf{J_N}][\mathbf{J_{N\text{-}1}}] \cdots [\mathbf{J_i}] \cdots [\mathbf{J_2}][\mathbf{J_1}] \right| = \begin{vmatrix} a_{11} & a_{12} \\ a_{21} & a_{22} \end{vmatrix} = a_{11}a_{22} - a_{12}a_{21} = 0 \tag{S16}$$

The resonance frequency equation in the above formula has a series of eigenvalues $\lambda_{in}\left(n = 1,\ 2,\ 3,\ \ldots\ r,\ \ldots\ n\ \ldots\right)$, which correspond to the nth eigenfrequency, respectively, so the rth resonance frequency can be expressed as follows.

$$\omega_{1r} = \cdots = \omega_{ir} = \cdots = \omega_{(N+1)r} = \lambda_{ir}^2 \sqrt{\frac{(YI)_i}{m_i l_i^4}} \tag{S17}$$

Section S2. The proof of the orthogonality of the modal function

Taking two different modes (rth and sth orders) of vibration response and substituting them into Equation (S3), respectively, the following equation can be obtained.

$$(YI)_i \frac{d^4\phi_{ir}(x)}{dx^4} = \omega_{ir}^2 m_i \phi_{ir}(x), \qquad (YI)_i \frac{d^4\phi_{is}(x)}{dx^4} = \omega_{is}^2 m_i \phi_{is}(x) \tag{S18}$$

Multiply the left and right sides of the above formula by $\phi_{ij}(x)$ and then integrate them in the beam length direction of the microbeams. Finally, after summing each microbeam, the following equation can be obtained. Where $j \in (r,s), i \in (1,2,3,...,N+1)$.

$$\begin{cases} \sum_{i=1}^{N+1} \left[\int_0^{x_i} \phi_{is}(x)(YI)_i \frac{d^4\phi_{ir}(x)}{dx^4} dx \right] = \sum_{i=1}^{N+1} \left[\omega_{ir}^2 \int_0^{x_i} \phi_{is}(x) m_i \phi_{ir}(x) dx \right] \\ \sum_{i=1}^{N+1} \left[\int_0^{x_i} \phi_{ir}(x)(YI)_i \frac{d^4\phi_{is}(x)}{dx^4} dx \right] = \sum_{i=1}^{N+1} \left[\omega_{is}^2 \int_0^{x_i} \phi_{ir}(x) m_i \phi_{is}(x) dx \right] \end{cases} \tag{S19}$$

The two equations in Equation (S19) are symmetrical. We do two partial integrations on the left side of the first equation and then use a similar processing method for the other one. Finally, we can get the following equation.

$$\sum_{i=1}^{N+1}\left[\begin{array}{l}\int_0^{x_i}\frac{d^2\phi_{is}(x)}{dx^2}(YI)_i\frac{d^2\phi_{ir}(x)}{dx^2}dx+\phi_{is}(x)(YI)_i\frac{d^3\phi_{ir}(x)}{dx^3}\Bigg|_0^{x_i}\\-\frac{d\phi_{is}(x)}{dx}(YI)_i\frac{d^2\phi_{ir}(x)}{dx^2}\Bigg|_0^{x_i}\end{array}\right]=\sum_{i=1}^{N+1}\left[\omega_{ir}^2\int_0^{x_i}\phi_{is}(x)m_i\phi_{ir}(x)dx\right] \quad \text{(S20)}$$

According to the boundary conditions in Equation (S8), the following relational formula can be obtained.

$$\sum_{i=1}^{N+1}\left[\phi_{is}(x)(YI)_i\frac{d^3\phi_{ir}(x)}{dx^3}\Bigg|_0^{x_i}-\frac{d\phi_{is}(x)}{dx}(YI)_i\frac{d^2\phi_{ir}(x)}{dx^2}\Bigg|_0^{x_i}\right]=0 \quad \text{(S21)}$$

Combining Equation (S20) and Equation (S21), and then simplifying, we can get the following equation. Using a similar method, we can also get Equation (S23).

$$\sum_{i=1}^{N+1}\left[\int_0^{x_i}\frac{d^2\phi_{is}(x)}{dx^2}(YI)_i\frac{d^2\phi_{ir}(x)}{dx^2}dx\right]=\sum_{i=1}^{N+1}\left[\omega_{ir}^2\int_0^{x_i}\phi_{is}(x)m_i\phi_{ir}(x)dx\right] \quad \text{(S22)}$$

$$\sum_{i=1}^{N+1}\left[\int_0^{x_i}\frac{d^2\phi_{ir}(x)}{dx^2}(YI)_i\frac{d^2\phi_{is}(x)}{dx^2}dx\right]=\sum_{i=1}^{N+1}\left[\omega_{is}^2\int_0^{x_i}\phi_{ir}(x)m_i\phi_{is}(x)dx\right] \quad \text{(S23)}$$

The above two equations can be subtracted and arranged to obtain the following relational formula.

$$\sum_{i=1}^{N+1}\left[\left(\omega_{ir}^2-\omega_{is}^2\right)\int_0^{x_i}\phi_{ir}(x)m_i\phi_{is}(x)dx\right]=0 \quad \text{(S24)}$$

Because $\omega_{ir}^2 \neq \omega_{is}^2$, the orthogonality of the mode shape function is as follows.

$$\sum_{i=1}^{N}\left[\int_{(i-1)l_0}^{il_0}\phi_{ir}(x)m_i\phi_{is}(x)dx\right]+\int_{l_b}^{l_b+l_m}\phi_{(N+1)r}(x)M_m\phi_{(N+1)s}(x)dx=\delta_{rs} \quad \text{(S25)}$$

In combination with Equation (S19), we can obtain another orthogonal condition of the mode function as follows.

$$\sum_{i=1}^{N}\left[\int_{(i-1)l_0}^{il_0}\phi_{ir}(x)(YI)_i\frac{d^4\phi_{is}(x)}{dx^4}dx\right]+\int_{l_b}^{l_b+l_m}\phi_{(N+1)r}(x)Y_mI_m\frac{d^4\phi_{(N+1)s}(x)}{dx^4}dx=\omega_{ir}^2\delta_{rs} \quad \text{(S26)}$$

Section S3. Bidirectionally coupled distributed parameter model and its steady-state solution under modal coordinates

Combining the mode function's orthogonality, mass normalization function, damping coefficient, vibration differential equation, and coupling circuit equation, the bidirectional coupled distributed parameter electromechanical model of the MEMS variable cross-section cantilever beam based piezoelectric vibration energy harvester under modal coordinates is obtained as follows.

$$\begin{cases} \dfrac{d^2\eta_r(t)}{dt^2}+2\zeta_r\omega_r\dfrac{d\eta_r(t)}{dt}+\omega_r^2\eta_r(t)+\Theta_r V(t)=F_r e^{i\Omega t} \\ C_p\dfrac{dV(t)}{dt}+\dfrac{V(t)}{R_l}-\sum_{r=1}^{\infty}\Theta_r\dfrac{d\eta_r(t)}{dt}=0 \end{cases} \tag{S27}$$

Next, the steady-state response solution of the distributed parameter electromechanical model under the simple harmonic excitation with the basic excitation $B(t)=B_0e^{i\Omega t}$ is solved. The amplitude of the acceleration of the basic excitation is $a_0=B_0\Omega^2$. According to the linear theory, the steady-state modal mechanical response and voltage response on steady-state load resistance of the variable cross-section piezoelectric cantilever beam are both simple harmonic quantities at the same frequency. Substituting $\eta_r(t)=H_re^{i\Omega t}$ and $V(t)=Ve^{i\Omega t}$ into formula (S27), the following equation can be obtained. Where $\Omega=2\pi f$ and $\omega_r=2\pi f_r$.

$$\begin{bmatrix} 4\pi^2\left(f_r^2-f^2+i2\zeta_r f_r f\right) & \Theta_r \\ -i2\pi f\sum_{r=1}^{\infty}\Theta_r & i2\pi fC_p+\dfrac{1}{R_l} \end{bmatrix}\begin{bmatrix} H_r \\ V \end{bmatrix}=\begin{bmatrix} F_r \\ 0 \end{bmatrix} \tag{S28}$$

Therefore, the steady-state solution of the bidirectionally coupled model is as follows. The steady-state modal mechanical response :

$$\eta_r(t)=\left[F_r-\frac{i2\pi f\Theta_r\sum_{r=1}^{\infty}\dfrac{\Theta_r F_r}{4\pi^2\left(f_r^2-f^2+i2\zeta_r f_r f\right)}}{\left(i2\pi fC_p+\dfrac{1}{R_l}\right)+i2\pi f\sum_{r=1}^{\infty}\dfrac{\Theta_r^2}{4\pi^2\left(f_r^2-f^2+i2\zeta_r f_r f\right)}}\right]\frac{e^{i2\pi ft}}{4\pi^2\left(f_r^2-f^2+i2\zeta_r f_r f\right)} \tag{S29}$$

The steady-state voltage response on load resistance :

$$V(t)=\frac{-i2\pi f\sum_{r=1}^{\infty}\dfrac{\Theta_r F_r}{4\pi^2\left(f_r^2-f^2+i2\zeta_r f_r f\right)}}{\left(i2\pi fC_p+\dfrac{1}{R_l}\right)+i2\pi f\sum_{r=1}^{\infty}\dfrac{\Theta_r^2}{4\pi^2\left(f_r^2-f^2+i2\zeta_r f_r f\right)}}e^{i2\pi ft} \tag{S30}$$

The relative displacement of the ith microbeam:

$$z_i(x,t)=\sum_{r=1}^{\infty}\left[F_r-\frac{i2\pi f\Theta_r\sum_{r=1}^{\infty}\dfrac{\Theta_r F_r}{4\pi^2\left(f_r^2-f^2+i2\zeta_r f_r f\right)}}{\left(i2\pi fC_p+\dfrac{1}{R_l}\right)+i2\pi f\sum_{r=1}^{\infty}\dfrac{\Theta_r^2}{4\pi^2\left(f_r^2-f^2+i2\zeta_r f_r f\right)}}\right]\frac{\phi_{ir}(x)e^{i2\pi ft}}{4\pi^2\left(f_r^2-f^2+i2\zeta_r f_r f\right)}\quad (i-1)l_0\le x\le il_0 \tag{S31}$$

The above-mentioned steady-state solution is a multi-modal solution, which can be applied to excitation at resonance frequencies as well as excitation at non-resonant frequencies. To get the maximum electrical response, the device generally works near the basic natural frequency or a certain

high-order natural frequency, that is $f \approx f_r$. At this time, the sum term of the above-mentioned steady-state solution is mainly determined by the rth order vibration mode.

Section S3. The relevant settings and attributes in the ANSYS modeling process.

The ANSYS finite element modeling process mainly includes: the establishment of geometric models, element and material attribute assignment of different modules, meshing, equipotential surface coupling, boundary condition setting and load application, model solving, etc. The properties of the elements in the modeling are: AlN piezoelectric layer uses SOLID 226 hexahedral elements, Si cantilever beams and mass blocks use SOLID 45 hexahedral elements, and load resistance uses CIRCU 94 elements for piezoelectric analysis. The trapezoidal cantilever beam used for model verification is a regular structure, and a mapping grid is used in the modeling process. Before solving, first couple all the nodes on the upper and lower surfaces of the AlN piezoelectric layer into equipotential surfaces. In the case of short circuit, the upper and lower surfaces are coupled into an equipotential surface, and in the case of open circuit, the upper and lower surfaces are each coupled into an equipotential surface. Under load conditions, the two nodes of the CIRCU 94 unit are coupled to the upper and lower surfaces of the AlN piezoelectric layer to form an electrical connection. Then set the boundary conditions and apply the load.

Table S1. Material properties of the beam and piezoelectric materials.

Parameter	Description	Value
ρ_b, ρ_p	Density of the Si beam substrate, the AlN piezoelectric layer	2329 kg/m^3, 3260 kg/m^3
Y_b, Y_p	Elastic modulus of the Si beam substrate, the AlN piezoelectric layer	170 Gpa, 350 Gpa
d_{31}	Strain coefficient of the AlN piezoelectric layer	-1.73 pC/N
$\varepsilon_{33}^{s} / \varepsilon_0$	Relative permittivity at a constant strain of the AlN piezoelectric layer	10.26

Table S2. Process flow of the MEMS trapezoidal cantilever beam based PVEH

	Process step name	Layer thickness	Two-dimensional cross-sectional view (A-A')
①	Prepare SOI wafer	$Si/SiO_2/Si$:<br>50 μm/1μm/350 μm	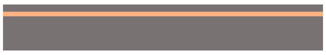
②	Thermal oxidation	SiO_2:200 nm	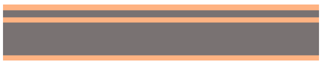
③	Bottom electrode preparation	Pt:120 nm	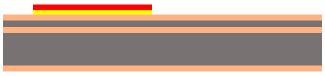
④	Growth of AlN film	AlN:1 μm	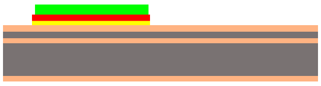
⑤	Top electrode preparation	Al:0.9 μm	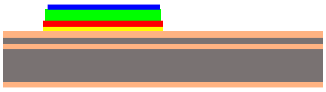
⑥	Front patterning	SiO_2:200nm, Al:100nm	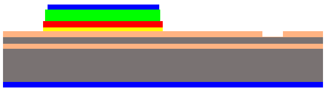
⑦	Front silicon etching	Si :50μm	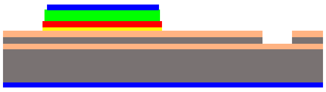
⑧	Form mass pattern	Al:100 nm	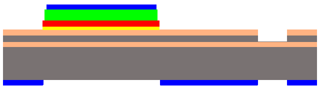
⑨	Back silicon deep-etching	Si:350 μm	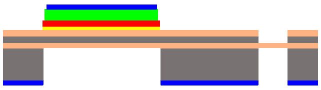
⑩	Structure release	SiO_2:1 μm	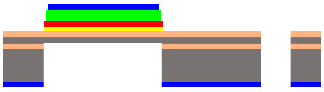

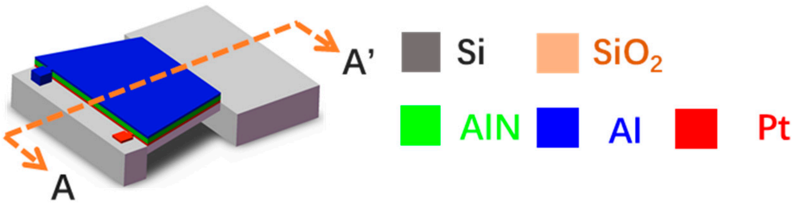

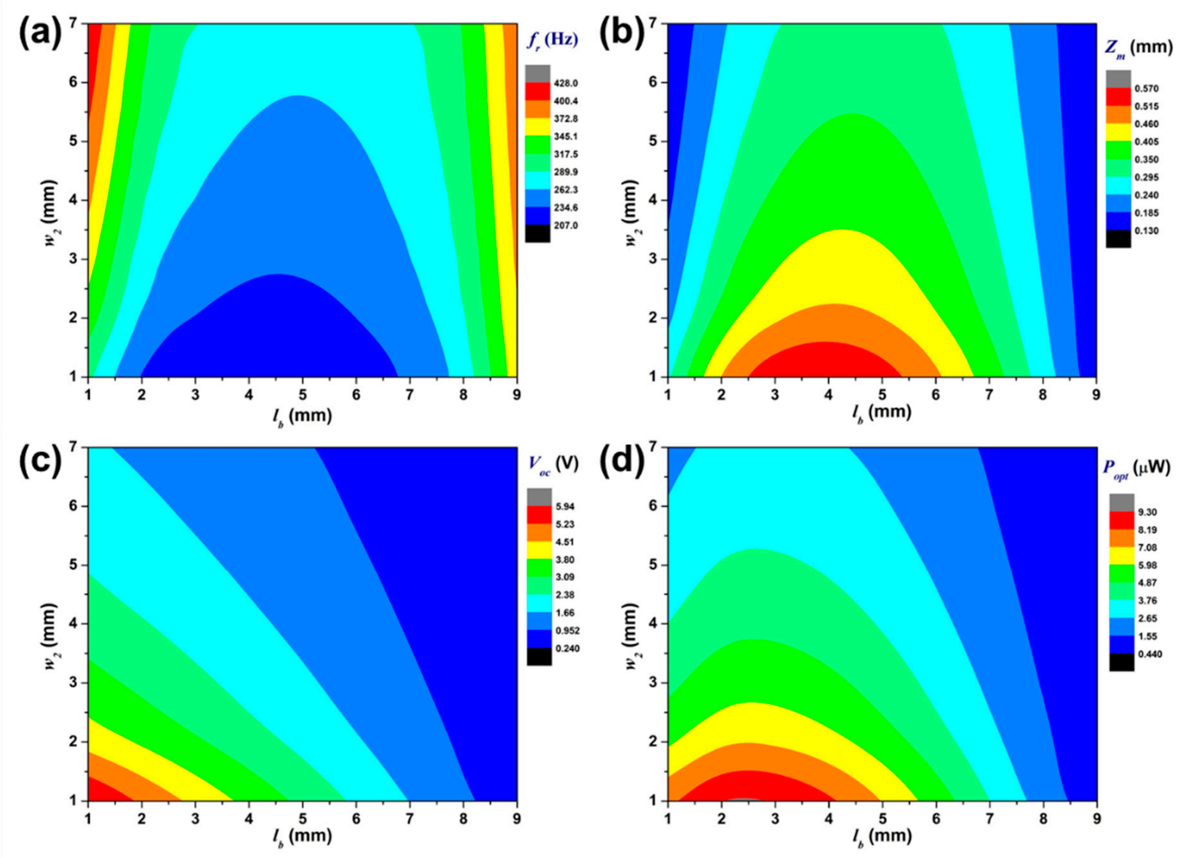

Figure S1. Contour maps of variations of the resonance frequency f_r (a), the maximum displacement of the tip mass z_m (b), the open-circuit voltage V_{oc} (c), and the optimal load output power P_{opt} (d) of the MEMS TCB based PVEH under different l_b and w_2.

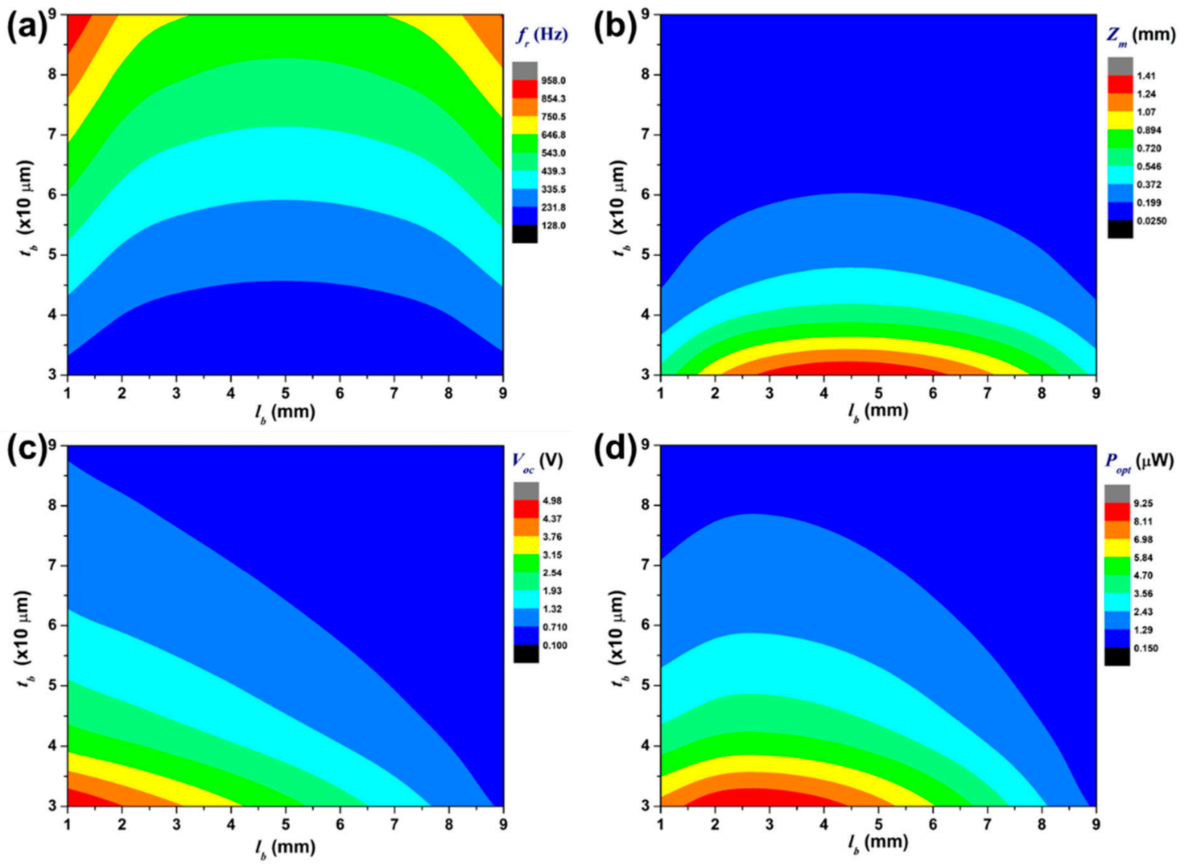

Figure S2. Contour maps of variations of the resonance frequency f_r (a), the maximum displacement of the tip mass z_m (b), the open-circuit voltage V_{oc} (c), and the optimal load output power P_{opt} (d) of the MEMS TCB based PVEH under different l_b and t_b.

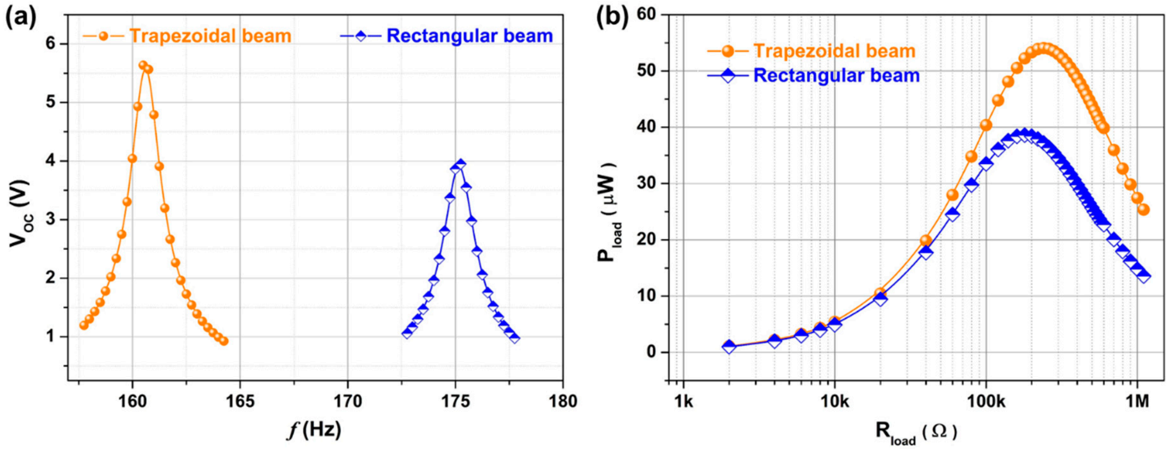

Figure S3. The open-circuit voltage frequency response curve (a) and the load output power curve (b) of the MEMS trapezoidal and rectangular beam based PVEH prototypes at the external excitation acceleration of 1g.

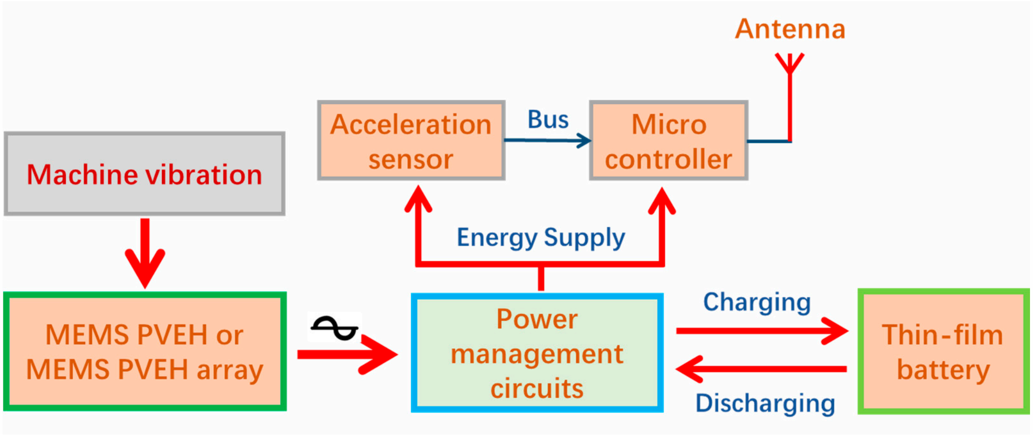

Figure S4. Schematic diagram of fault monitoring node based on the MEMS PVEH.
